# In Situ X-Ray Study During Thermal Cycle Treatment Combined with Complementary Ex Situ Investigation of InGaN Quantum Wells

**DOI:** 10.3390/nano15020140

**Published:** 2025-01-17

**Authors:** Ewa Grzanka, Sondes Bauer, Artur Lachowski, Szymon Grzanka, Robert Czernecki, Byeongchan So, Tilo Baumbach, Mike Leszczyński

**Affiliations:** 1 Institute of High Pressure Physics, Polish Academy of Sciences, Sokolowska 29/37, 01-142 Warsaw, Poland; 2 TopGaN Ltd., Solec 24/90, 00-403 Warsaw, Poland; 3 Institute for Photon Science and Synchrotron Radiation, Karlsruhe Institute of Technology, Hermann-von-Helmholtz-Platz 1, 76344 Eggenstein-Leopoldshafen, Germany; 4 Competence Center for III-Nitride Technology, C3NiT-Janzén, Solid State Physics and NanoLund, Lund University, P.O. Box 118, 22100 Lund, Sweden; 5 Laboratory for Applications of Synchrotron Radiation, Karlsruhe Institute of Technology, Kaiserstr. 12, 76131 Karlsruhe, Germany

**Keywords:** MOVPE, InGaN QWs, in situ X-ray diffraction, annealing, indium homogenization, thermal treatment, indium fluctuation, decomposition

## Abstract

In situ X-ray reciprocal space mapping was performed during the interval heating and cooling of InGaN/GaN quantum wells (QWs) grown via metal–organic vapor phase epitaxy (MOVPE). Our detailed in situ X-ray analysis enabled us to track changes in the peak intensities and radial and angular broadenings of the reflection. By simulating the radial diffraction profiles recorded during the thermal cycle treatment, we demonstrate the presence of indium concentration distributions (ICDs) in the different QWs of the heterostructure (1. QW, bottom, 2. QW, middle, and 3. QW, upper). During the heating process, we found that the homogenization of the QWs occurred in the temperature range of 850 °C to 920 °C, manifesting in a reduction in ICDs in the QWs. Furthermore, there is a critical temperature (*T* = 940 °C) at which the mean value of the indium concentration starts to decrease below 15% in 1. QW, indicating the initiation of decomposition in 1. QW. Moreover, further heating up to 1000 °C results in extended diffuse scattering along the angular direction of the diffraction spot, confirming the propagation of the decomposition and the formation of trapezoidal objects, which contain voids and amorphous materials (In-Ga). Heating InGaN QWs up to *T* = 1000 °C led to a simultaneous decrease in the indium content and ICDs. During the cooling phase, there was no significant variation in the indium concentrations in the different QWs but rather an increase in the defect area, which contributes to the amplification of diffuse scattering. A comparison of ex situ complementary high-resolution transmission microscopy (Ex-HRTEM) measurements performed at room temperature before and after the thermal cycle treatment provides proof of the formation of four different types of defects in the QWs, which result from the decomposition of 1. QW during the heating phase. This, in turn, has strongly influenced the intensity of the photoluminescence emission spectra without any detectable shift in the emission wavelength λ_MQWs_.

## 1. Introduction

The effects of thermal stress on the structure and optical properties of InGaN/GaN multiple quantum wells (MQWs) are relevant for a deep understanding of temperature-induced structural and chemical modifications. The latter have been investigated via ex situ X-ray diffraction and electron microscopy methods [1,2,3,4,5,6,7,8,9,10,11,12]. Several phenomena, such as the interdiffusion of indium and gallium in MQWs [3,8,9], phase separation [5], indium aggregation, segregation [2,11,13,14,15], and the decomposition [6] of InGaN/GaN MQWs, were revealed after annealing. It has been reported that annealing in the temperature range from 800 °C to 900 °C led to the confinement of indium-rich clusters near the InGaN wells, while further annealing above 950 °C induced indium fluctuations and disturbances in the QW structures [2,7]. This confinement was attributed to the diffusion of the indium atoms of the In-rich phase within and across the QWs, which led to an increase in the indium content in the nominal QWs [3,8,9]. Furthermore, the degradation of the QWs was attributed to the formation of In-rich precipitates induced via phase separation after thermal annealing [3,5,8]. Smalc-Koziorowska et al. [16] used transmission electron microscopy (TEM) to reveal the formation of trapezoidal voids due to the agglomeration of migrating metal vacancies. These trapezoidal objects in the QW degraded areas comprising voids partly filled with crystalline In and an amorphous material surrounded by a rim of high-In-content In_x_ Ga_1−x_N.

Previous ex situ studies, dedicated to investigating the annealing of InGaN QWs, required a patch of samples that were individually subjected to thermal stress at specific temperatures. However, to date, no information has been published on structural changes during the cooling phase of the thermal cycle. Moreover, there is no clear consensus as to whether modifications in the structure of InGaN/GaN MQWs during the heating phase are reversible. Modifications in the structure of MQWs due to annealing were demonstrated at the nanometer scale, and changes that occurred during the intermediate stages could not be visualized through ex situ investigations. This has prompted several researchers to perform non-destructive in situ X-ray studies to visualize the structural evolution of the grown layers and their interface qualities via time-resolved investigations, for instance, during the growth of GaN-based materials [17,18,19,20,21,22,23,24,25].

In fact, in situ X-ray reflectivity and in situ X-ray crystal truncation rod methods have been employed to characterize interfaces and measure grown film thickness during the MOVPE growth of a single InGaN/GaN QW under various growth conditions [18,19,20]. Furthermore, in situ XRD reciprocal space map measurement was proven to be beneficial for determining the periodicity, composition, and strain of GaN/AlGaN superlattices [22] and InGaN films during the MOVPE growth process [24].

In this work, InGaN/GaN MQW samples underwent interval heating and cooling in a series of steps. Each step had a duration of *t_A_* = 15 min, during which the temperature was kept constant. During this step, the acquisition of reciprocal space maps (RSMs) lasted for *t_M_* = 5 min. The investigation was based on interval heating and cooling to follow the evolution of the structure and detect the critical temperatures for the homogenization and decomposition of the InGaN/GaN MQWs during heating. Additionally, our in situ X-ray diffraction study (in situ XRD) aimed to provide information on the unknown structural modifications during the cooling phases and compare the indium distribution contents of the individual QWs at the same temperatures depicted in the heating and the cooling phases of the thermal cycle treatment. We also compare the microstructure and photoluminescence properties (measured ex situ) of the pristine and thermally treated QW heterostructures.

## 2. Materials and Methods

### 2.1. MOVPE Growth and Annealing

**Figure 1:**
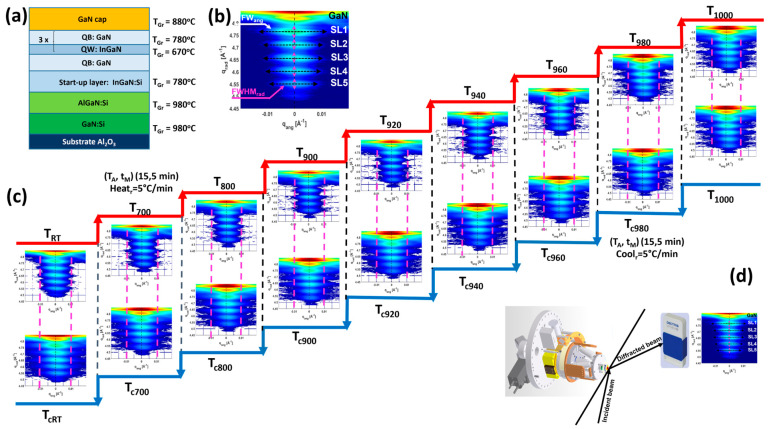
(**a**) Layout of the InGaN QW sample. (**b**) Example of the reciprocal space map for the (0004) reflection showing the GaN(0004) reflection and the InGaN satellite peaks, where the full angular broadening (*FW_ang_*) and radial full-width half-maximum (*FWHM_rad_*) are illustrated by a horizontal black dashed line and vertical magenta line, respectively. (**c**) RSMs recorded during the heating and cooling phases with the indication of annealing time *t_A_* and the acquisition time *t_M_*. (**d**) Schematic presentation of the in situ X-ray diffraction set-up including the hexapod for mounting and aligning the sample and the one-dimensional Mythen detector.

The investigated structure was grown in an Aixtron close-coupled showerhead (CCS 3x2”). As substrates, we used 2 μm thick GaN templates on sapphire with a 300 nm thick AlGaN layer and 7.5% Al content. To grow thick layers, trimethylgallium (TMGa), trimethylaluminum (TMAl), and ammonia (NH_3_) were used as gallium, aluminum, and nitrogen precursors, respectively. To grow GaN quantum barriers and InGaN layers, triethylgallium (TEGa), trimethylindium (TMIn), and ammonia (NH_3_) were used as gallium, indium, and nitrogen precursors, respectively. Start-up InGaN layers with an indium content of 3.5% were grown at 760 °C, followed by three-fold quantum wells (MQWs) grown in nitrogen as a carrier gas at a pressure of 333 mbar with a N:III ratio of 25,000. Quantum wells (QWs) were grown at a temperature of 670 °C and a growth rate of 0.6 nm/min, while the quantum barriers (QBs) were grown at a temperature of 780 °C, with a growth rate of 1 nm/min. The MQWs were capped with a 50 nm-thick GaN overlayer. The substrate temperature was controlled using a thermocouple placed under the graphite susceptor and confirmed via whole-wafer temperature mapping using an AIXTRON instrument (AIXTRON, Herzogenrath, Germany) [26]. The layout of the sample is presented in Figure 1a.

### 2.2. In Situ X-Ray Diffraction Analysis

The sample was annealed in a nitrogen atmosphere in a chamber coupled with a heavy-duty diffractometer at the NANO beamline at the Karlsruhe KARA synchrotron facility in Germany. The sample was mounted on a hexapod inside the chamber for alignment with respect to the incident beam (see Figure 1d). The sample holder was heated with a laser diode from the back side, and the temperature was controlled with the thermocouple and via a laser pyrometer, using a feedback loop for temperature stabilization. In situ X-ray measurements were performed at an energy of *E* = 14.9 keV and a wavelength of *λ* = 0.8321 Å using a linear Mythen detector (Dectris, Baden, Switzerland) with 1280 channels and a channel size of 50 µm (see Figure 1d). The detector–sample distance was 1039 mm, which gave an angular resolution of 0.00275 deg. Reciprocal space maps (RSMs) of the GaN(0004) reflection were recorded at different temperature steps during the heating phases (herein *RT*, *T*_700_, *T*_800_, *T*_900_, *T*_920_, *T*_940_, *T*_960_, *T*_980_, and *T*_1000_) and the cooling phases *T_c_*_980_, *T_c_*_960_, *T_c_*_940_, *T_c_*_920_, T_c900,_ *T_c_*_800_, *T_c_*_700_, and *T_cRT_* (see Figure 1c). RSMs were acquired by keeping the Mythen detector at a fixed Bragg angle position corresponding to the diffraction angle of the GaN(0004) reflection and rocking the sample, which was vertically mounted, and the chamber was rotated around the vertical axis to incline the sample with a diffraction angle of the GaN(0004) reflection. Furthermore, using a high reflection order gave us an advantage in resolving the diffraction peaks, since the separation distance Δ*Q_z_* between the peaks in the case of the GaN(0004) reflection is twice that of the GaN(0002) reflection. Additionally, there was no need to use a filter system to avoid the saturation of the detector at the main GaN peak, as is the case for GaN(0002). However, the use of this filter also has the disadvantage of reducing the intensities of the satellite peaks.

The RSM acquisition lasted *t_M_* = 5 min, while the sample was annealed for *t_A_* = 15 min (see Figure 1c). During the heating phase (and, alternatively, the cooling phase), while adjusting from one temperature to another, for example, from *T*_700_ to *T*_800_, a heating rate of 10 °C/min was used. Meanwhile, the increase in temperature between *T*_920_ and *T*_940_ was performed at a heating rate of 5 °C/min (see Figure 1c).

From the different RSMs corresponding to the measured temperatures mentioned above, radial at *Qz* ≠ 0 Å^−1^ and *Qx* = 0 Å^−1^ and angular (i.e., at *Qz* = *Q_max_*, *Q_x_* ≠ 0 Å^−1^), diffraction profiles were derived that also corresponded to the vertical and horizontal cuts of the RSMs.

The evaluation of the radial diffraction profiles was based on fitting the curves using the Origin 2018b 64Bit software package [27] with the Pseudo-Voigt function and deriving the peak areas, referred to here as the *A_T_* (heating phase) of the satellites and the *A_Tc_* of the cooling phase, as well as the full-width half-maximum (*FWHM*), termed *FWHM_rad_*. To estimate the angular broadening due to diffuse scattering, which is termed *FW_ang_*, we measured the width of the angular profiles corresponding to the 1% of maximum peak intensity *I_max_* (see Figure 1b). An example of the fitting procedure using the Pseudo-Voigt function for the sample at the *T_cRT_* state after the thermal cycle treatment is shown in Figure S1a in the Supplementary Information (SI). In this fitting, the baseline is indicated by a dashed line for the case of the 0th order and SL1, whereby the integrated area was calculated above the background delimited by the drawn baseline. A non-linear square fit algorithm was applied using the Origin software package, and the best fit was indicated by the chi-square (chi^2^) value closest to 1.

Additionally, we simulated the radial diffraction profiles using the Leptos 7.9 software package “DIFFRAC^plus^ LEPTOS 7” from the company Bruker, Karlsruhe, Germany [28]. The model used in Leptos was based on the two-wave approximation for the dynamical diffraction theory and a recursive matrix formalism [28]. The entire stack of layers forming the structure of the sample (illustrated in Figure 1a) was considered in the simulation model.

The simulation, applied for all the radial diffraction profiles recorded during heating and cooling, was based on the assumption of an indium concentration distribution (ICD) that could be different for each quantum well, 1. QW (bottom QW), 2. QW (middle QW), and 3. QW (upper QW), between the minimum *χ_min_* and maximum *χ_max_* values. The simulation model of the superlattice proposed by the Leptos 7.9 software package offers the option of considering the quantum well layer as an ensemble of *N* sublayers (*N* = 10 is the grading in our case) in which the indium concentration *χ* is distributed following either linear, sinusoidal, or exponential profiles delimited by the values *χ_min_* and *χ_max_*. The selected profile was sinusoidal, and the mean value was simply determined as follows: <*χ_in_*> = (*χ_min_* + *χ_max_*)/2.

The simulation model applied in the Leptos software package also assumes that all the interfaces are sharp. For verification purposes, simulations with a roughness parameter *R* at different interfaces were performed, and the R values converged to zero for the best-fitting results.

Additionally, we made the assumption that InGaN quantum wells are fully strained to the GaN, meaning that no strain relaxation was considered. These assumptions were confirmed by measuring asymmetric reflections of the orders (105), (106), and (107) at room temperature (not presented here), which show that the GaN and InGaN reflections are underlined on the same horizontal wave vector *Qx* coordinates.

The fitting parameters used for modeling are *χ_min_* and *χ_max_*, the thicknesses of the QWs (*d*_1._ *_QW_*, *d*_2._ *_QW_*, and *d*_3._ *_QW_*), and the GaN barriers (*d*_1*GaN*_, *d*_2*GaN*_, and *d*_3*GaN*_). We also derived the mean values of the indium concentration <*χ_in_*> (*T*) = (*χ_min_* + *χ_max_*)/2 as a function of the temperature for the different InGaN QWs. In addition, we used the indium concentration *χ_in_* of the start-up layer InGaN-Si as a fitting parameter to better fit the SL0 peak.

An exemplary demonstration of the simulation and the resulting fitting is given for the *T_cRT_* state in Figure S1b of the SI, where the complete diffraction profile was fitted by the model.

It should be noted that the SL0 peak, which is to the left of the GaN(0004) peak, is also influenced by the indium concentration *χ_in_* of the start-up layer InGaN-Si, while the peak to the right of the GaN(0004) corresponds to the Al_0.08_GaN (see Figure 1a).

### 2.3. Transmission Electron Microscopy (TEM)

Microscopic structural changes caused by the thermal cycle were investigated via scanning and high-resolution transmission electron microscopy, STEM, and HRTEM, respectively. Cross-sectional TEM images were obtained using an FEI TECNAI G^2^ F20 S-TWIN microscope manufactured by Rigaku in Japan, operated at 200 kV and equipped with a Fischione Model 3000 high-angle annular dark-field (HAADF) STEM detector (Fischione Instruments, Export, PA, USA). The indium concentration χ*_In_STEM_* in the quantum wells was mapped with an approach developed by Rosenauer et al. [29]. The TEM specimens were prepared via mechanical polishing using diamond-lapping films followed by Ar^+^ ion milling at 4 keV, with a final step at 400 eV.

### 2.4. Photoluminescence (PL) Properties

The optical properties of the samples before (*T_RT_*) and after the thermal treatment cycle (*T_cRT_*) were determined using photoluminescence (PL). The spectra were measured at room temperature (*RT*), and excitation was obtained using a 325 nm He-Cd laser, operating in continuous-wave operation mode. The PL was recorded using an Acton SpectraPro500i Spectrometer and CCD camera (Acton Research Corporation, Acton, MA, USA) with a Si detector, which was air-cooled down to −21 °C.

## 3. Results and Discussions

### 3.1. In Situ Study of Structural Modifications in QWs During Heating Phase

**Figure 2:**
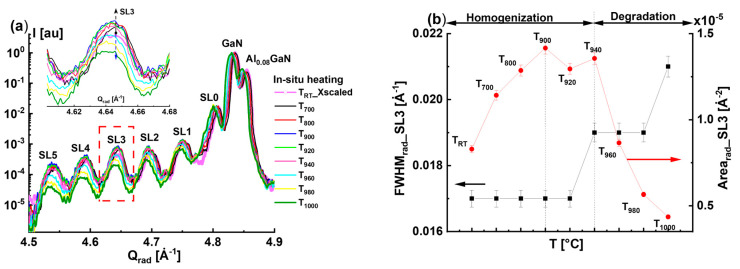
(**a**) Comparison of the radial diffraction profiles as a function measured during the heating phase at *T_RT_*, *T*_700_, *T*_800_, *T*_900_, *T*_920_, *T*_940_, *T*_960_, *T*_980_, and *T*_1000_. The XRD profile of *T_RT_* was rescaled along the X-axis. The inset corresponds to the radial profile of satellite peak SL3, indicated by a dashed square. (**b**) Variation in the full-width half-maximum *FWHM_rad__SL3* (left axis) and the area of satellite peak SL3 *Area_rad__SL3* (right axis) with the temperature.

The radial diffraction profiles of the GaN(0004) reflection, which correspond to the heating phases (*RT*, *T*_700_, *T*_800_, *T*_900_, *T*_920_, *T*_940_, *T*_960_, *T*_980_, and *T*_1000_), are compared in Figure 2a. For better clarity, all the radial diffraction profiles were normalized to the peak intensity of GaN. Furthermore, the points between *T_RT_* and T_1000_ are arranged on a non-linear *X*-scale to better track the changes, especially in the range [*T*_900_–*T*_1000_]. The inset particularly presents the changes in the radial diffraction profiles of satellite peak SL3, which first appears as an increase in the peak intensity when the temperature increases to *T*_940_ and, then, a decrease in the peak intensity as the temperature exceeds *T*_960_. To visualize this effect, we had to rescale the *T_RT_* profiles along the X-axis knowing that the shift in the peak position is correlated to the thermal expansion coefficient. From the fitting of the diffraction profiles using the Origin software package, we precisely determined the *Q_z_* coordinates of the GaN(0004) reflections that were used to calculate the out-of-plane lattice parameter *c*(T), as a function of the temperature, and the thermal expansion coefficients. These values are given in Table S1 in the Supplementary Information (SI).

To track the changes in the radial diffraction profiles of the InGaN satellite peaks during the heating phase, we focused on the variations in the peak area (*Area_rad__SL3*) and *FWHM_rad__SL3* belonging to satellite peak SL3 as a function of the temperature. In Figure 2b, we distinguish three different regions. For the temperature range *T* below *T*_900_, the *Area_rad__SL3* increases with the temperature, while the *FWHM_rad__SL3* remains the same. Therefore, we could deduce that heating the QW samples to *T*_900_ induces an enhancement in the coherent diffracted intensities of SL3. This could also be attributed to the thermal diffusion of the indium atoms from the indium-rich clusters (IRCs) into the nominal QWs, which compensates for the out-going indium atoms diffusing from the InGaN QWs to the surrounding matrix, including the GaN barriers. The latter behavior could explain the increase in the SL area peak intensities, a finding well in accordance with the results of Chuo et al. [3,9].

**Figure 3:**
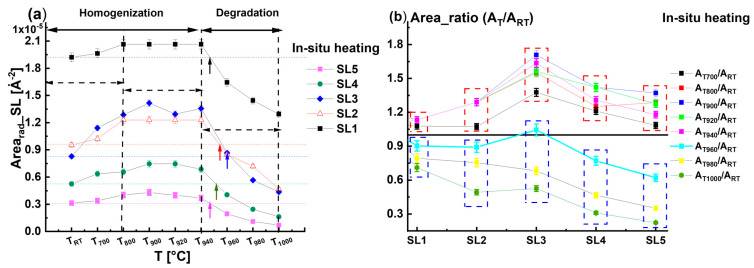
(**a**) Variation in the area of the radial profiles *Area_rad_* corresponding to all satellites recorded during the heating phase. (**b**) The area ratio *A_T_/A_RT_* at different temperatures *A_T_* with respect to room temperature *A_RT_* as a function of the satellite peak order.

There is a slight variation in the *Area_rad__SL3* in the temperature range *T*_900_ to *T*_940_ which was accompanied by the *FWHM_rad__SL3* starting to increase. In the final stage of the heating phase (*T* > *T*_940_), there is a decrease in the *Area_rad__SL3*, indicating a worsening of the structure quality due to the decomposition of the InGaN QWs beginning without knowing exactly which QW is affected. This also affects the *FWHM_rad__SL3*, which also continues to increase, indicating the enhancement of the indium fluctuation of the InGaN QWs. The simultaneous decrease in the *Area_rad__SL3* and increase in the *FWHM_rad__SL3* could be interpreted as the consequence of thermal diffusion of the indium and gallium atoms from the InGaN QWs towards the InGaN/GaN interfaces. A similar phenomenon was reported by Kusakabe et al. [1]. The variation in *Area_rad__SL* with temperature for all satellites (SL1, SL2, SL3, SL4, and SL5) shown in Figure 3a confirms that the beginning of the InGaN decomposition occurs when the temperature exceeds *T*_940_. We found that SL1, SL2, SL4, and SL5 behave similarly to SL3. The horizontal dashed lines in Figure 3a indicate that the satellite peak area *Area_rad__SL* corresponds to the room temperature *T_RT_* prior to the heating procedure. The drawn arrows indicate the temperature at which the *Area_rad__SL* falls lower than *T_RT_*. First, we can conclude that the decrease in the *Area_rad__SL* occurs for temperatures higher than *T*_940_. However, the loss in *Area_rad__SL* with respect to the *T_RT_* states is different for every satellite, and we can see that *Area_rad__SL5* becomes lower than the value of *T_RT_* at *T* = 945 °C. Meanwhile, for *Area_rad__SL4*, the loss occurred rather at *T* = 955 °C. To demonstrate the loss in peak intensity of the diffraction profiles with respect to the initial state of *T_RT_*, we calculated the radial diffraction area ratios *A_TT_/A_RT_* of the different satellite peaks for all the temperatures (i.e., *A_T_*_700_*/A_RT_*, *A_T_*_800_*/A_RT_*, *A_T_*_900_*/A_RT_*, *A_T_*_920_*/A_RT_*, *A_T_*_940_*/A_RT_*, *A_T_*_960_*/A_RT_*, *A_T_*_980_*/A_RT_*, and *A_T_*_1000_*/A_RT_*). The variation in these ratios with temperature for all satellite peaks (SL1, SL2, SL3, SL4, and SL5) is shown in Figure 3b. We have underlined the ratio *A_TT_/A_RT_* = 1 with a horizontal solid line. For all the satellites, the *A_TT_/A_RT_* ratios are below one when the temperature *T* ≥ *T*_960_.

For temperature *T* < *T*_960_, the ratios *A_TT_/A_RT_* are higher than 1, meaning that homogenization of the indium distribution has taken place, enhancing the radial diffraction profile areas *Area_rad__SL*. Furthermore, the *Area_rad__SL* decreases with the satellite peak order for a temperature *T* higher than *T*_960_, while it increases with the SL order for a *T* below *T*_960_. And SL3 presents the satellite with the maximum intensity (see Figure 3b). The initiation of the InGaN QWs degradation via heating the sample to a temperature higher than *T*_940_ was found in accordance with the finding of Lin et al. [2], who attributed the worsening of the QW structures to the disappearance of the regular indium-rich clusters.

**Figure 4:**
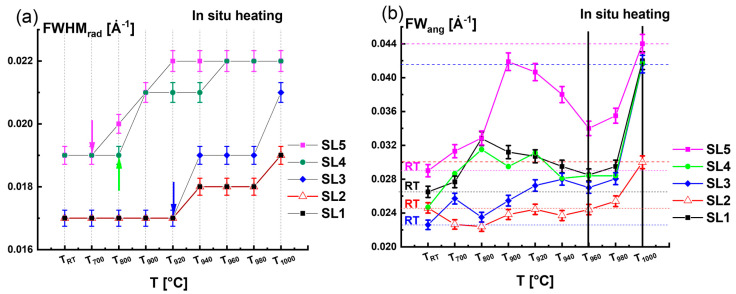
(**a**,**b**) Variation in radial *FWHM_rad_* and angular broadening *FW_ang_* with the temperature, respectively, during the heating phase for all satellite peaks.

The changes in the *FWHM_rad_* and *FW_ang_* during the heating phase were elaborated as a function of the temperature and depended on the satellite order. The variations in the *FWHM_rad_* and *FW_ang_* with temperature are given in Figure 4a and Figure 4b, respectively. Figure 4a shows a gradual increase in the *FWHM_rad_* during the heating phase. The starting point of the *FWHM_rad_* increase differs between the satellite peaks. It is worthwhile to note that the higher-order satellite peaks SL4 and SL5 are more sensitive to the changes in the indium distribution in the InGaN quantum wells in comparison to the lower-order satellite peaks like SL1, SL2, and SL3. To enhance the visibility of sensitivity, we have inserted arrows in Figure 4a, which indicate the starting point of the increase in *FWHM_rad_*. For SL4, *FWHM_rad_* starts to increase at *T*_800_, while for the case of SL5, it starts at *T*_700_. In contrast, for the lower-order satellite peak SL1, the *FWHM_rad_* varies at higher temperatures *T* > *T*_920_.

To clearly visualize changes in the angular broadening *FW_ang_* with the temperature, two dashed magenta lines are drawn on the RSMs in Figure 1c corresponding to *FW_ang_* = 0.02 Å^−1^. From the RSMs in Figure 1c, the angular broadening due to diffuse scattering exceeds the dashed lines as *T* rises higher than *T*_960_. Furthermore, the variation in the *FW_ang_* with *T* is plotted for SL1, SL2, SL3, SL4, and SL5 in Figure 4b.

For a better understanding, we depicted the *FW_ang_* at *RT* with horizontal dashed lines for the different satellites in Figure 4b. Up to *T*_960_, depending on the satellite order, the *FW_ang_* either slightly increases (e.g., SL1, SL2, and SL3) or increases strongly with some fluctuation (e.g., SL4 and SL5). However, there is a clear tendency for a fast increase in the *FW_ang_* values as the temperature exceeds *T*_960_ due to defect formation, which extends the diffuse scattering around the SL diffraction spots. It should be emphasized that the remarkable increase in the diffuse scattering is well correlated with the decrease in the *Area_rad__SL* of the InGaN QW coherent diffracted intensity (Figure 3a).

If we compare the initial state at *RT* and the end state at *T*_1000_ during the heating phase of the QWs, we note that the area ratio (*A_T_*_1000_/*A_RT_*) is equal to 0.7 for SL1 and 0.2 for SL5 (see green hexagon symbol plotted in Figure 3b) confirming the loss of coherent diffracted intensities at *T*_1000_ due to the decomposition of indium in the InGaN QWs. This consequently led to an augmentation of the radial broadening *FWHM_rad_* from *FWHM_rad_* = 0.017 Å^−1^ to *FWHM_rad_* = 0.022 Å^−1^, as shown in Figure 4a and by a doubling of the angular broadening *FW_ang_* value because of the defect formation.

### 3.2. In Situ Study of Structural Changes in QWs During the Cooling Phase

In this chapter, we examine the modifications in the QW structures during cooling from *T*_c1000_ to room temperature *T_cRT_*. Importantly, the states of the QWs at room temperature prior to heating and after cooling are termed *T_RT_* and *T_cRT_*, respectively. It is worth noting that the starting and end states of the cooling phase correspond to *T_c_*_1000_ and *T_cRT_*, respectively. For a better understanding, it is useful to remember that the QWs at T1000 contain a proportion of decomposed QWs, as confirmed by the extended diffuse scattering and the loss of coherent diffracted SL peak intensities. As a consequence, the decrease in the area ratio (*A_T_/A_RT_*) represents a reliable indicator to detect the critical temperature for the degradation of the InGaN QWs.

**Figure 5:**
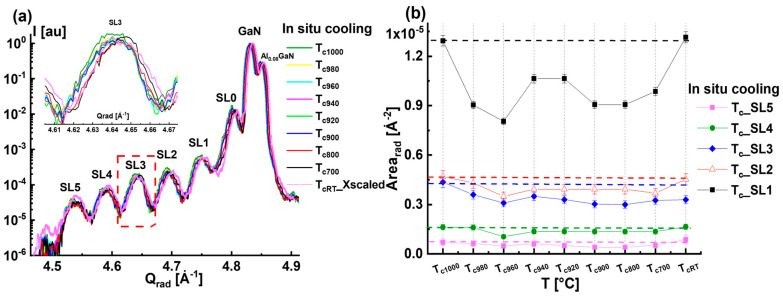
(**a**) Comparison of the radial diffraction profiles as a function measured during the cooling phase from *T_c_*_1000_ to *T_cRT_*. The XRD profile of *T_cRT_* was rescaled along the X-axis. The inset corresponds to the radial profile of SL3, as indicated by a dashed square. (**b**) Variation in peak area *Area_rad__SL* of the radial profiles of all satellites with the temperature recorded during the cooling phase.

Figure 5a presents a comparison of the radial diffraction profiles that were recorded during the cooling from *T_c_*_1000_ to *T_cRT_*. The *T_cRT_* curve was scaled along the X-direction. For clarity, the inset in Figure 5a shows only the diffraction profiles of SL3, which do not reveal a significant variation in comparison with the one reported during the heating phase (see Figure 2a and Figure 5a). Figure 5b shows the variation in the satellite peak areas *Area_rad__SL* as a function of temperature *T*. The different dashed horizontal lines refer to the *Area_rad__SL* of the starting state *T*_1000_. The overall behavior of *Area_rad__SL* with temperature for the different SLs consists of a slight decrease as the samples cool to *T_c_*_940_ and then an increase to recover the loss of intensities at *T_cRT_* and again achieve similar values as in the *T*_1000_ state. As a result, the *Area_rad__SL* of *T_c_*_1000_ and *T_cRT_* are comparable for all satellites (see Figure 5b).

**Figure 6:**
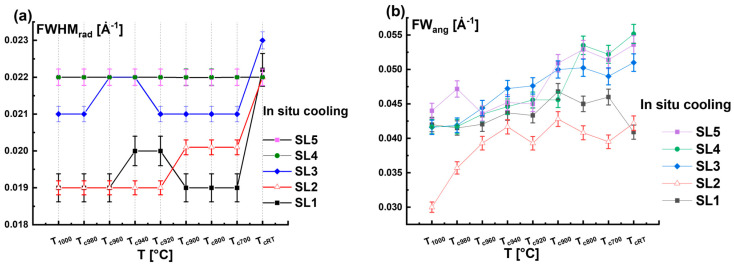
(**a**,**b**) Variation in the radial *FWHM_rad_* and the angular broadening *FW_ang_* with the temperature, respectively, during the cooling phase for the different satellite peaks.

To better understand the structural modifications during the cooling phase, *FWHM_rad_* and *FW_ang_* are plotted in Figure 6a and Figure 6b, respectively, as a function of the temperature from *T_c_*_1000_ up to *T_cRT_*. We deduce that the radial broadening *FWHM_rad_* (Figure 6a) remains the same, which indicates the absence of reversibility in the behavior, i.e., homogenization and decomposition of the QWs during the heating phase. However, we can observe an increase in the angular broadening *FW_ang_* (Figure 6b), most likely resulting from the decomposed InGaN QWs, representing defect sites in the sample and generating an additional diffuse scattering along the angular direction. In fact, *FW_ang_* has increased from about 0.042 Å^−1^ at *T_c_*_1000_ up to 0.052 Å^−1^ at *T_cRT_* (c.f. Figure 6b). In conclusion, the structure of the QWs at the cooled state *T_cRT_* differs from the *T_c_*_1000_ state mainly in terms of the extension of the diffuse scattering along the angular direction, resulting from the enlargement of InGaN QW areas that had already decomposed during the heating process when the temperature exceeded *T*_960_.

**Figure 7:**
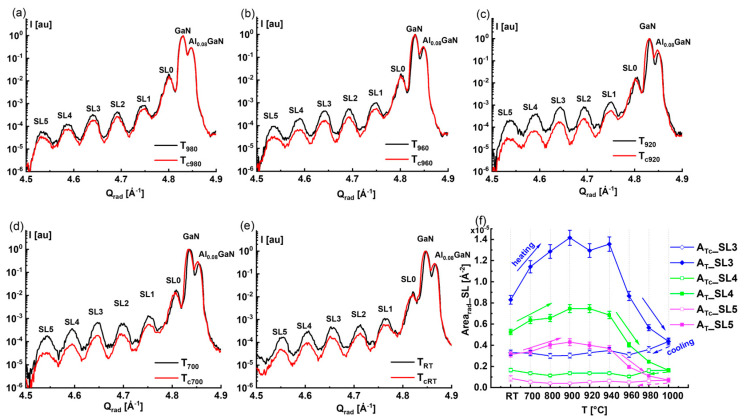
Plots (**a**–**e**) compare the radial diffraction profiles recorded during the heating and the cooling phases of the thermal cycle treatment at specific temperatures, such as *T* = 980 °C, 960 °C, 920 °C, 700 °C, and *RT*, respectively. (**f**) Variation in the radial peak area *Area_rad__SL* with the temperature T during the complete thermal cycle treatment for higher-order satellite peaks SL3, SL4, and SL5.

To compare the structure of the QWs at specific temperatures corresponding to the heating and cooling states, Figure 7a–e simultaneously illustrate the radial diffraction profiles of the samples at the selected temperatures.

All the curves in Figure 7a–e are normalized to the GaN peak of the GaN(0004) reflections to mainly focus on the intensity variation corresponding to the satellites. It is clear that the curves of the heating and cooling states increasingly diverge from each other as the temperature varies from *T*_1000_ to *T_RT_*. This permits us to conclude that there was no recovery of the decomposed InGaN QWs during the cooling phase.

Figure 7f shows the *Area_rad__SL* with *T* for higher-order satellites SL3 and SL4 during the heating and cooling cycles. The results confirm that the degradation of QWs occurs when the sample is heated beyond *T*_940_, as the *Area_rad__SL* drops with temperature. There is no reversibility in the crystal structure and the indium distribution between the heating and cooling cycling of the QWs as the coherent diffracting structure decreases (see arrows in Figure 7f).

**Figure 8:**
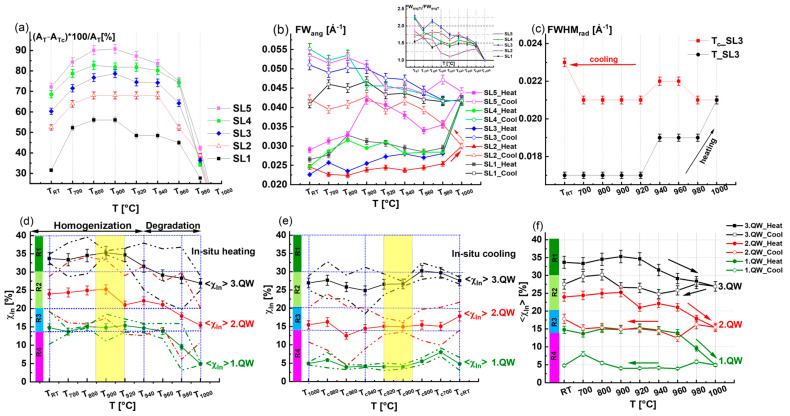
(**a**) The variation in the percentage loss (A_T_-A_Tc_)*100*/A_T_* of diffracted peak intensities of all satellites with the temperature; (**b**) variation in the angular broadening *FW_ang_* with temperature T during the complete thermal cycle treatment for all satellite peaks; (**c**) variation in the radial broadening *FWHM_rad_* as a function of the temperature for SL3; (**d**) variation in the indium concentration *χ_In_* [%] of 1. QW, 2. QW, and 3. QW with temperature during the heating (**e**) and cooling phases of the thermal cycle treatment. (**f**) Dependence of indium mean value concentration <χ_In_> on the temperature of 1. QW, 2. QW, and 3. QW during the thermal cycle treatment. Ranges: *R*1 = 30%< *χ_In_* < 40%; *R*2 = 20%< *χ_In_* < 30%; *R*3 = 14%< *χ_In_* < 20%; *R*4 = 0%< *χ_In_* < 14%. The vertical discontinuous blue lines refer to *T_RT_*, *T*_940_, *T*_1000_, *T_c_*_940_, and *T_cRT_*.

The loss in intensity at a specific temperature was evaluated by determining the percentage difference in *Area_rad__SL* between the heating *A_T_* and the cooling *A_Tc_* (*A_T_-A_Tc_*) with respect to *A_T_*, as shown in Figure 8a. The percentage loss values (A_T_-A_Tc_)*100*/A_T_* derived from the values in Figure 7f indicate the loss of diffracted peak intensity for all of the satellites. As the peak areas *A_Tc_* changed slightly with the temperature during cooling, the curves behaved similarly to those during heating. The highest percentage loss of intensity is found at SL5, with the maximum value of 90% at *T*_900_. This indicates that the higher-order satellites are more affected than the lower-order satellites, where the percentage loss is about 55% at *T*_900_ (c.f. Figure 8a). Additionally, Figure 8b compares the variation in *FW_ang_* with the *T* corresponding to the heating (solid symbols) and cooling phases (open symbols) of the thermal cycle treatment for different SLs. We deduce that *T*_940_ (indicated by a vertical dashed blue line) is a critical temperature beyond which *FW_ang_* starts to increase, indicating the start of the decomposition of the InGaN QWs. The enlargement of *FW_ang_* continues by further heating up to *T*_1000_ and during the cooling of the sample.

This supports the formation of defects such as voids, precipitates, etc., resulting from the InGaN QW decomposition in the annealed sample at a T higher than *T*_940_, which in turn, induced an extension of the diffuse scattering measured via *FW_ang_*. These kinds of defects were revealed via HRTEM by Smalc-Koziorowska et al. in InGaN QWs after annealing [16].

The onset in Figure 8b corresponds to the ratio *FW_angTc_/FW_angT_* as a function of *T*. By comparing the *FW_ang_* at specific temperatures, we find that the ratio varies between 1.5 and 2 for the different SLs, with the exception of SL5. There is no reversible behavior in the *FW_ang_* due to the formation of defects coming from the presence of indium precipitation after the InGaN QW decomposition.

Figure 8c compares the evolution of *FWHM_rad_* for SL3 with the temperatures during the heating and cooling phases. The amplification of *FWHM_rad_* during the heating phase and the reachable value at *T*_1000_ were more or less retained during the cooling process. Furthermore, the behavior of *FWHM_rad_* with *T* was found to be irreversible during cooling, similar to *Area_rad__SL*. As a result, the *FWHM_rad_* values are higher during the cooling process than during the heating phase (see Figure 8c).

In summary, if we compare the QW samples at their initial state after the MOVPE growth at *T_RT_* and after undergoing a complete thermal cycle treatment at *T_cRT_*, we can reliably confirm the manifestation of InGaN decomposition in the QWs at a *T* higher than *T*_940_. This was proven through the loss of coherently diffracted peak intensities of the different InGaN satellites and via the amplification of the radial broadening *FWHM_rad_* due to the increase in the indium distribution. Simultaneously, it was also confirmed via the enhancement of the angular broadening *FW_ang_* that resulted from the defect build-up in the decomposed proportion of QWs.

The variation in *Area_rad__SL* with temperature *T* has served as a good and trustworthy indicator to inform the grower about the quality improvement of QWs via annealing, in terms of indium homogenization in QWs when the *Area_rad__SL* has increased. Moreover, it also enabled us to detect the critical temperature for the decay of the SL peak intensities as we heated the sample above *T*_940_ (see Figure 2b and Figure 3a).

The initiation of decomposition and the appearance of the resulting defects like voids, precipitates, and amorphous compounds in the InGaN QWs were revealed through the expansion of diffuse scattering around the GaN(0004) main spot of the satellite peaks during the thermal cycle treatment (see Figure 4b, Figure 6b and Figure 8b). However, it was fairly hard to determine the variation in the indium concentration distribution (ICD) and to localize the defect formation in the individual quantum wells 1. QW, 2. QW, and 3. QW without simulating the diffraction curves.

As mentioned above in Section 2.2, “In-situ X-ray diffraction analysis”, the simulation of the diffraction curves was performed using the Leptos software package “DIFFRAC^plus^ LEPTOS 7” from the company Bruker, Karlsruhe, Germany [28]. In our chosen simulation model, the indium concentration distribution (ICD) of each quantum well was described via a sinusoidal profile in the range of [*χ_min_*, *χ_max_*]. From the results of best fitting the measured diffraction profile with the simulation model (see Figure S1a), we derived the minimum, maximum, and mean values of the indium concentrations *χ_min_*, *χ_max_*, and <*χ_In_*> = (*χ_min_* + *χ_max_*)/2, respectively. Figure 8d and Figure 8e, respectively, correspond to the variation in indium concentration *χ_In_* (*T*) with temperature during the heating and cooling phases. The mean value of the indium concentration <*χ_In_*> (*T*) plots are indicated using a solid green hexagon for the bottom quantum well 1. QW, a solid red circle for the middle quantum well 2. QW, and a solid black square for the upper quantum well 3. QW. For the different <*χ_In_*> (*T*) curves corresponding to the three quantum wells, 1. QW, 2. QW, and 3. QW, there are two related dashed curves that are drawn below and above the symbol <*χ_In_*> (*T*) curves and refer to the minimum and maximum indium concentrations *χ_min_* and *χ_max_*, respectively. In Figure 8d and Figure 8e, the extent of the indium concentration distribution (ICD) in the individual QWs, namely 1. QW, 2. QW, and 3. QW, is limited by the two discontinuous *χ_min_* (*T*) and *χ_max_* (*T*) curves.

**Figure 9:**
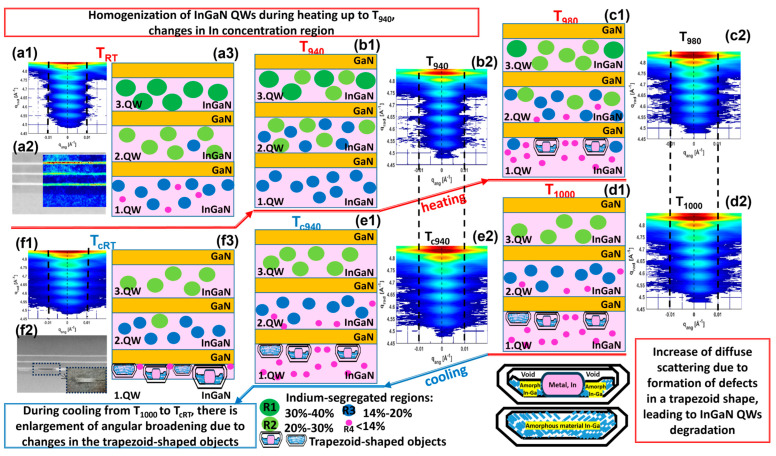
Reciprocal space maps of (0004) reflection of the InGaN heterostructure at (**a1**) *T_RT_*, (**b2**) *T*_940_, (**c2**) *T*_980_, (**d2**) *T*_1000_, (**e2**) *T_c_*_940_, and (**f1**) *T_cRT_*. (**a2**) STEM image (left) together with indium concentration map (right) for InGaN QWs at *T_RT_*; (**f2**) STEM image for InGaN QWs at *T_cRT_*, schematic presentation of the indium concentration distribution (ICD) given by ranges *R*1 = 30% < *χ_in_* < 40%, *R*2 = 20% < *χ_in_* < 30%, *R*3 = 14% < *χ_in_* < 20%, and *R*4 = *χ_in_* < 14% for 1. QW, 2. QW, and 3. QW at (**a3**) *T_RT_*, (**b1**) *T*_940_, (**c1**) *T*_980_, (**d1**) *T*_1000_, (**e1**) *T_c_*_940_, and (**f3**) *T_cRT_*, together with illustrations of the defect indicated by trapezoid-shaped objects.

To distinguish between the high, medium, and low indium concentration regions in the individual quantum wells 1. QW, 2. QW, and 3. QW, we introduce in Figure 8d–f the ranges *R*1 = 30% < *χ_In_* < 40%, *R*2 = 20% < *χ_In_* < 30%, *R*3 = 14% < *χ_In_* < 20%, and *R*4 = *χ_In_* < 14%, indicated by the vertical colored bars parallel to the Y-axis. As shown in Figure 8d, during the heating phase, we found that the extent of the ICD becomes narrow at 850 °C < *T* < 920 °C, indicating the occurrence of 1. QW, 2. QW, and 3. QW homogenization (regions are denoted by yellow rectangles). Furthermore, the ICD increases as the *χ_min_* (*T*) and *χ_max_* (*T*) curves start to diverge again in the temperature range from *T*_940_ to *T*_980_. Regarding the cooling phase given by Figure 8e, the ICD again becomes narrow in the range of *T_c_*_920_ to *T_c_*_850_ as the sample cools to *T_cRT_* (see yellow rectangular). It can be concluded that the temperature range *T_c_*_920_ to *T_c_*_850_ is suitable for achieving reduced ICDs with minimum fluctuation in coherent parts of the QWs. To better understand the indium concentration regions existing in the different QWs, we suggest focusing on specific temperatures, such as *T_RT_*, *T*_940_, and *T*_1000_, as indicated by the blue vertical dotted lines in Figure 8d, as discussed below:

- Composition of InGaN QWs at RT: The upper QW (3. QW) contains InGaN zones with indium concentrations *χ_in_* of R1. We can see that the ICD is comprised inside the R1 region, while the middle QW is in the stack. The middle QW (2. QW) comprises InGaN regions with indium concentrations *χ_in_* of *R*2 and *R*3, as the ICD curves are crossing these two regions. Finally, the bottom QW (1. QW) contains regions with indium concentrations *χ_in_* of *R*3 and *R*4 (see Figure 8d). The composition of InGaN QW at *RT* is schematically illustrated in Figure 9(a3), where the regions *R*1, *R*2, *R*3, and *R*4 are indicated by dark-green, light-green, blue, and magenta circles, respectively;
- Composition of InGaN QWs at *T*_940_: 3. QW contains zones with indium concentrations *χ_in_* of *R*1 and *R*2 as the lower *χ_min_* is crossing the range *R*2 and *χ_max_* is going through the R1 region, and 2. QW involves zones with indium concentrations χ_in_ of R2 and R3 regions. Moreover, 1. QW includes indium concentrations *χ_in_* of *R*3, with *χ_in_* ≅ 16%, and *R*4, with *χ_in_* ≅ 14%, at *T*_940_. The composition of InGaN QWs at *T*_940_ is exemplified in Figure 9(b1), where the regions *R*1, *R*2, *R*3, and *R*4 are indicated by dark-green, light-green, blue, and magenta circles, respectively;
- Composition of InGaN QWs at *T*_1000_: 3. QW contains areas with indium concentrations *χ_in_* of only *R*2, while 2. QW includes the regions with *χ_in_* of *R*3 and *R*4. Additionally, 1. QW comprises regions of indium concentrations *χ_in_* of mostly *R*4 with *χ_in_* ≅ 5%, which corresponds to the lowest indium concentration as a result of the decomposition of 1. QW at *T*_1000_. The degradation of 1. QW, determined from the modeling, was demonstrated by a significant reduction in the indium concentration *χ_in_* and attributed to the formation of trapezoidal objects into 1. QW at *T*_1000_, the latter being pictured in Figure 9(c1,d1) by drawing trapezoidal objects in the lower quantum well 1. QW, which probably comprises voids and amorphous material (In-Ga), as previously argued regarding similar heterostructures [16]. A detailed description of these objects will be presented in Section 3.3. Furthermore, the possible formation of these voids and defects was confirmed by the enlargement of the diffuse scattering in the RSM of GaN(0004), as shown in Figure 9(c2,d2), corresponding to *T*_980_ and *T*_1000_.

In summary, it would be possible to describe the transformation in each individual QW during the heating phase, as follows.

For the upper quantum well 3. QW, as the temperature increases from *T_RT_* to *T*_940_, there is a decrease in the formation of region *R*1, with high indium concentrations of 30% < *χ_in_* < 40%, and region *R*2, with indium concentrations of 20% < *χ_in_* < 30%, which leads to a mixture of indium concentrations at *T*_940_ in 3. QW. By further heating the sample, the indium concentration of *R*3 disappears, and regions with an *χ_in_* of *R*2 remain, whereby the *χ_in_* decreases from 25% to 20% as the T increases from *T*_940_ to *T*_1000_.

For the middle quantum well 2. QW, at *T_RT_*, 2. QW represents a mixture of zones with an *χ_in_* of *R*2 and *R*3. When the temperature increases from *T_RT_* to *T*_940_, 2. QW still consists of regions with an *χ_in_* of *R*2 and *R*3. By further heating, the regions with an *χ_in_* of *R*2 = 20% < *χ_in_* < 30% are transformed into an *χ_in_* of *R*3 = 14% < *χ_in_* < 20% and *R*4 = *χ_in_* < 14%

For the lower quantum well 1. QW, by heating the sample from *T_RT_* to *T*_940_, 1. QW includes a mixture of indium concentrations *χ_in_* of *R*3 and *R*4. By further heating toward *T*_1000_, the regions with an *χ_in_* of R3 are transformed into regions with an *χ_in_* of *R*4.

The evolution of the indium concentration *χ_in_* during the cooling phase is shown in Figure 8e, where the blue vertical dotted lines refer to *T*_1000,_ *T_c_*_940_, and *T_cRT_*. For a better understanding, it is worthwhile to analyze the compositions of the individual QWs at these specific temperatures.

For the composition of InGaN QWs at *T_c_*_940_, 3. QW mainly contains regions with an indium concentration *χ_in_* of *R*2 = 20% < *χ_in_* < 30%, while 2. QW consists of two indium concentration regions, *R*3 and *R*4. Finally, 1. QW comprises only regions with an indium concentration *χ_in_* of R4. It should be noted that the ICD of the QWs became narrow as the sample was cooled from *T*_1000_ to *T_c_*_940_.

For better clarity, the distribution of indium concentrations at *T_c_*_940_ is schematically presented in Figure 9(e1), where the regions *R*2, *R*3, and R4 are indicated by light-green, blue, and magenta circles, respectively.

For the composition of InGaN QWs at *T_cRT_*, we note that the extent of the ICD is reduced to 2% for 3. QW and to 3% for 1. QW after the thermal cycle treatment. The indium concentration for the 3. QW *χ_in_* is about 27.4% of region *R*2, while it is *χ_in_* ≅ 4.7% for the 1. QW corresponding to region *R*4. However, the extent of the ICD is a little bit larger for 2. QW and is about 7%, but it still contains only the concentration of region *R*3 = 14% < *χ_in_* < 20%. The configuration describing the InGaN heterostructures at the final stage after the thermal cycle treatment is elucidated in Figure 9(f3), where the QW heterostructures are characterized by less indium fluctuation, as confirmed by the reduced ICD.

It is very important to simultaneously compare the InGaN heterostructures at specific temperatures during the heating and cooling phases such as (*T_RT_*, *T_cRT_*) in Figure 9(a1–a3,f1–f3) and *T*_940_ (*T*_940_, *T_c_*_940_) in Figure 9(b1,b2,e1,e2). Furthermore, Figure 8f compares the behavior of <*χ_in_*> (*T*) during heating and cooling and shows a decrease in the indium concentration for all the QWs during cooling due to the decomposition phenomenon preceded by the quantum well homogenization process. It should be emphasized that <*χ_in_*(*T*)> does not vary as strongly during the cooling phase as in the heating phase. If we compare the mean values for the indium concentration <*χ_in_*> of the different QWs at *T_RT_* and *T_cRT_*, we deduce that <*χ_in_*> has moved from *R*1 to *R*2 for 3*. QW*, from R2 to R3 for 2. QW, and from *R*3 to *R*4 for 1. QW. Similar behavior is found when we compare the <*χ_in_*> between *T*_940_ and *T_c_*_940_.

### 3.3. Comparison of Microstructure and Optoelectronic Properties of InGaN QWs at Room Temperature Before and After Thermal Cycle Treatment

**Figure 10:**
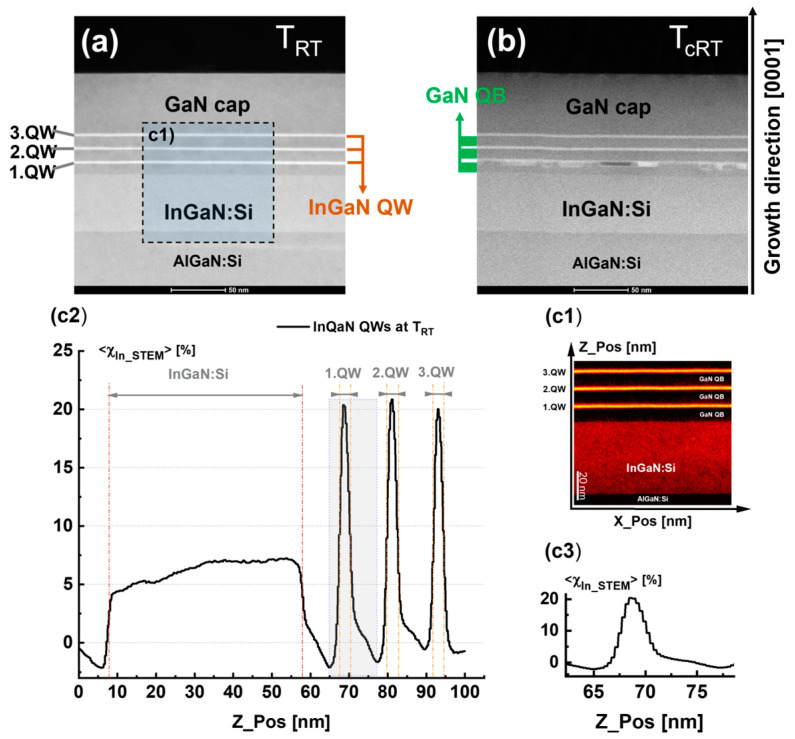
STEM images of QW heterostructures corresponding to (**a**) T_RT_ after the MOVPE growth and (**b**) at T_cRT_ after thermal cycle treatment; the scale bar is 50 nm. (**c1**) Indium concentration map with a size of 100 × 100 nm in InGaN QW heterostructures derived from STEM at T_RT_ prior to the thermal cycle treatment, (**c2**) the indium concentration <χ_In-STEM_> profiles derived from the map of (**c1**), and (**c3**) <χ_In-STEM_> profiles of 1. QW corresponding to the dashed box in (**c2**) and showing the presence of an indium concentration distribution (ICD) in the individual QWs.

Figure 10a,b compare the microstructures of the InGaN QW heterostructures examined at *T_RT_* after MOVPE growth and after thermal cycle treatment at *T_cRT_*, respectively. The STEM image in Figure 10a of the *T_RT_* state reveals MQWs with good crystal quality, which are free of defects. These MQWs are surrounded by an InGaN layer at the bottom and a GaN cap layer from the top. Moreover, we observe sharp interfaces between QWs and QBs on the bottom and upper sides. The thickness of the QWs is determined to be around 3 nm using TEM. It varies between 3 and 4 nm for different individual QWs.

A detailed analysis of the indium concentration in 1. QW, 2. QW, and 3. QW, based on the Rosenauer method [29], was performed only for the QWs at *T_RT_*. An indium concentration map <*χ_In-STEM_*> with a scale of 100 nm × 100 nm, which was derived from STEM images, is presented in Figure 10(c1). From this map, the <*χ_In-STEM_*_>_ profile was first determined by averaging along a sample thickness of about 100 nm, which corresponds to the “Y_pos” transverse direction to “X_pos”, and then integrating the indium concentration over the “X_pos” direction for each “Z_pos”, as shown in Figure 10(c2).

This map reveals two important features: (i) differences in the mean value of the indium concentration <*χ_in-STEM_*> for 1. QW, 2. QW and 3. QW and (ii) the presence of segregated indium regions corresponding to different concentration regions in accordance with the results derived from modeling the diffraction curves shown in Figure 8f. However, we found that the mean indium concentration value, which was derived for the XRD simulation corresponding to the whole InGaN QW sample at TRT, is <*χ_in_*> ≅ 15% for 1. QW, ≅ 25% for 2. QW, and 28% for 3. QW. These values are not exactly the same as the ones derived from the map of 100 × 100 nm (i.e., <*χ_In-STEM_*> ≅ 20% for 1. QW, 2. QW, and 3. QW) (see Figure 8f and Figure 10(c2)). This difference could be explained by the ability of the XRD analysis to provide more statistical determination for the indium content over several microns, in comparison with the local 100 × 100 nm map derived from STEM images. Furthermore, the profile <*χ_In-STEM_*> in Figure 10(c3) demonstrates the presence of an indium concentration distribution (ICD) in individual QWs rather than a constant indium concentration.

To better understand the reason for the variation in the <*χ_in_*> for the different QWs, it is worth reporting on the MOVPE growth conditions. In fact, InGaN QWs were grown via MOVPE at an extremely low temperature, 670 °C, in order to obtain QWs with an indium concentration <*χ_in_*> above 20% for green emission. However, due to the difference between the in-plane lattice parameters of the GaN buffer layer and the grown InGaN QW, the in-plane strain at this interface prevents the achievement of a desirable indium content for 1. QW, which had the lowest indium incorporation of <*χ_in_*> ≅ 15%, with an improvement in the indium incorporation for the subsequent 2. QW (<*χ_in_*> ≅ 25%) and 3. QW (<*χ_in_*> ≅ 28%). This would explain the observed increase in the indium concentration, as determined by the in situ X-ray analysis, which demonstrated a high <*χ_in_*> for 3. QW, an intermediate <*χ_in_*> for 2. QW, and a lower <*χ_in_*> for 1. QW. Regarding the QWs at *T_cRT_*, the STEM image in Figure 10b reveals a decomposed 1. QW with extent defect regions over the QW and the presence of dark contrast regions, while 2. QW and 3. QW remain in good quality and with well-defined interfaces.

**Figure 11:**
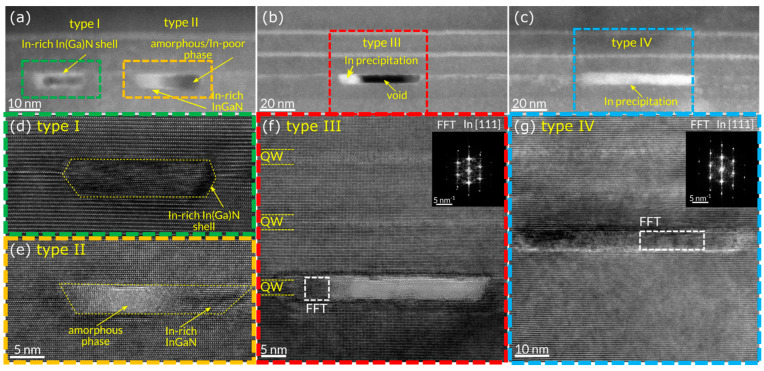
TEM images of different types of defects in 1. QW at T_cRT_ corresponding to different locations of the QW. (**a**) Types I and II are highlighted by green and orange boxes, respectively. (**b**) Defect type III is indicated by a red box and (**c**) defect type IV is indicated by a blue box. The corresponding HRTEMs of different defect types: (**d**) type I of the green box, (**e**) type II of the orange box, (**f**) type III of the red box, and (**g**) type IV of the blue box.

Figure 11 corresponds to the TEM and HRTEM of the InGaN QW heterostructures at TcRT after the thermal cycle treatment. Figure 11a–c depict different locations in the TEM cross-section, which are differentiated by four different types of defects in 1. QW, labeled according to types I, II, III, and IV.

The HRTEM of defect type I, which corresponds to the green box in Figure 11a, is shown in Figure 11d and reveals hexagonal objects with In-rich In(Ga)N shells and interior voids, while the HRTEM images in Figure 11e, which is the cropped orange box in Figure 11a, indicate that type II defects are formed in a trapezoidal object with an amorphous phase inside and In-rich InGaN. Moreover, in different regions in 1. QW, we found another, type III, which is indicated by a red box in Figure 11b and is shown at high resolution in Figure 11f, where it is possible to clearly see the formation of a trapezoidal void with In precipitation next to it and an In-rich In(Ga)N shell. In addition, a type IV defect, which is illustrated by the blue rectangle in Figure 11c and shown in Figure 11g, shows indium precipitation in a trapezoidal object.

Furthermore, the size of the defects varies from a few nanometers (type I) up to 50nm (type IV). We deduce that these four types of defects are different states of the object detected in the HRTEM images. We can assume that, due to the lateral diffusion of indium and gallium atoms at elevated temperatures, defect type I can be transformed into an intermediate form as defect type II, and finally, it led also to the formation of defect type III.

Our structural study, which was based on combining in situ XRD and ex situ HRTEM investigations, demonstrates important modifications in the indium concentration after the completion of thermal cycle treatment. This induced a change in the composition of the QW heterostructures, which is expected to strongly influence their photoluminescence properties (PL).

**Figure 12:**
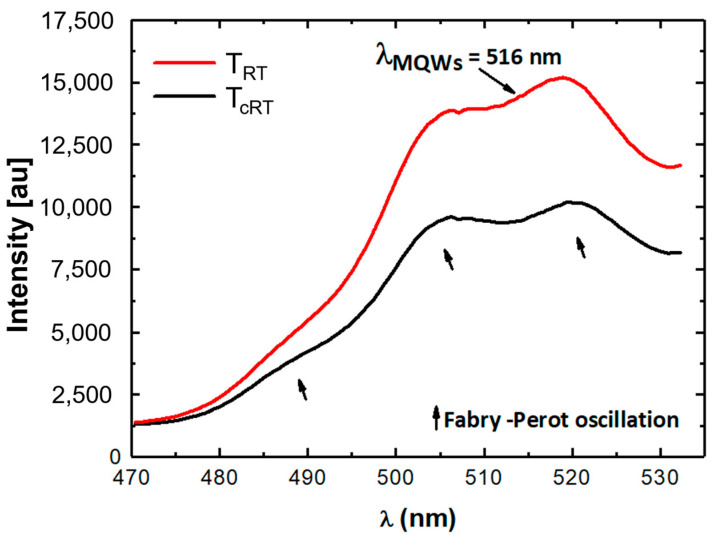
Comparison of the emission spectra of the InGaN QW heterostructures recorded before and after the thermal cycle treatment T_RT_ and T_cRT_, respectively.

Figure 12 shows a comparison of the PL spectra at *T_RT_* and *T_cRT_*. The PL measurements reveal an emission wavelength *λ_MQWs_* of around 516 nm for the as-grown sample measured in the *T_RT_* state. A similar emission *λ_MQWs_* value was obtained at *T_cRT_* after the completion of the thermal cycle treatment. It should be pointed out that the emissions spectra recorded at *T_RT_* and *T_cRT_* are broad around the wavelength *λ_MQWs_* of 516 nm, which reflects the presence of an indium concentration distribution (ICD) and is in accordance with the results derived from the simulation of the XRD curves (see Figure 8d–f) and from the chemical map determined from the TEM image in Figure 10c, where the ICD varies for 1. QW, 2. QW, and 3. QW at *T_RT_* and *T_cRT_*.

The broadening of the emission spectra could also be attributed to the thickness fluctuation of *QWs/QBs* and to the expected threading dislocation that can form in quantum well heterostructures grown on a sapphire template.

Moreover, the intensity of the spectrum measured at *T_cRT_* after the thermal treatment is about 60% lower than the one recorded prior to the thermal cycle. This can be attributed to the decomposition of 1. QW, which is proven via HRTEM (Figure 11) and the simulation shown in Figure 8d–f and is schematically illustrated above in Figure 10.

Due to Fabry–Perot oscillation (which is due to the sapphire substrate) and yellow luminescence close to the emission from QWs, peak fitting was applied to precisely determine the *FWHM_PL_* of the PL signals, as well as the peak positions. For the sample at *T_RT_*, we obtain *FWHM_PL_* = 44 ± 2 nm and *λ_MQWs_* = 516 nm. After thermal treatment at *T_cRT_*, the *FWHM_PL_* increases to 52 ± 2 nm, while the peak position remains the same at *λ_MQWs_* = 516 nm.

## 4. Conclusions

The aim of this study was to use an in situ X-ray investigation to determine the missing information related to changes in the InGaN QW crystal structure at different intermediate temperatures during heating and cooling. The indium concentration distribution of the different QWs at high temperatures, including *T*_700_, *T*_800_, *T*_900_, *T*_920_, *T*_940,_ and *T*_1000_, was determined by simulating the diffraction curves of GaN 400, which was derived from the acquired RSMs with a high resolution at the NANO beamline synchrotron facility KARA. In our opinion, the investigation of eight samples heated to specific temperature values via an ex situ investigation at room temperature cannot fully describe the QWs at these temperatures since the samples underwent additional modification during the cooling phase. This study demonstrates the effectiveness and importance of using an in situ analysis to replace a patch of eight samples that were separately heated to *T*_700_, *T*_800_, *T*_900_, *T*_920_, *T*_940,_ *T*_960_, *T*_980_, and *T*_1000_ and then cooled down to *T_RT_* for an ex situ examination. In this work, we developed an analysis procedure to control the overall quality of the InGaN QW heterostructures that involved the peak intensities, as well as the radial and angular broadening, *FWHM_rad_* and *FW_ang_*, determined by fitting the satellite peaks SL1, SL2, SL3, SL4, and SL5. This method proved sufficient for following changes in the QWs in terms of homogenization and to detect the critical temperature for the onset of QW degradation, which is about *T* = 940 °C. However, it was not possible to separately explore the structural modification in terms of the indium concentration distribution in each QW. Therefore, the dependence of the satellite peak areas *Area_rad_*, *FWHM_rad_*, and *FW_ang_* on temperature during thermal cycle treatment can be considered a reliable tool for the grower to detect the temperature that induces transformations in QW heterostructures. For a clear understanding of the indium content fluctuations inside the lower 1. QW, middle 2. QW, and upper 3. QW, it was crucial to simulate all diffraction curves with the assumption of the indium concentration distribution (ICD) in the QWs and with abrupt interfaces to obtain the best fit of the measured radial diffraction profiles. This enabled us to determine the compositions of the individual quantum wells 1. QW, 2. QW, and 3. QW for all the intermediate temperatures crossed during the thermal cycle treatment in terms of the following well-defined ranges of indium concentration: *R*1 = 30% < *χ_in_* <40%, *R*2 = 20% < *χ_in_* <30%, *R*3 = 14% < *χ_in_* <20%, and *R*4 = *χ_in_* <14%.

In summary, the structural changes occurring during thermal cycle treatment were schematically translated into a graphical abstract, given in Figure 9, which enables the comparison of the ICDs of the QWs at specific temperatures during the heating and cooling phases based on the quantification of the indium concentration derived from the simulation of the in situ X-ray diffraction curves.

The RSMs demonstrated an extension in diffuse scattering around the GaN(0004) diffraction spots generated by the formation of defects in the QWs as the temperature exceeded *T*_960_. Diffuse scattering was found to be remarkably enhanced as the sample was cooled to *T_cRT_*. The HRTEM explicitly demonstrated the formation of four types of defects in 1. QW. Type I appeared as hexagonal objects with In-rich In(Ga)N shells and voids, while type II was a trapezoidal object with an interior amorphous phase and In-rich InGaN. Type III corresponded to a trapezoidal void with indium precipitation surrounded by an In-rich In(Ga)N shell. Finally, defect type IV appears as indium precipitation in a trapezoidal form.

The photoluminescence properties, which were measured at room temperature before and after the thermal cycle treatments *T_RT_* and *T_cRT_*_,_ were visibly affected by the changes in the ICD of the QWs’ composition during the thermal treatment. The broad behavior of the emissions spectra was in accordance with the different ICDs revealed in 1. QW, 2. QW, and 3. QW, and the loss of intensity of the PL curve was interrelated with the decomposition of 1. QW and the formation of defects that was indicated via HRTEM.

## Data Availability

The data presented in this study are available upon request from the corresponding authors.

## Supplementary Materials

The following supporting information can be downloaded at www.mdpi.com/article/10.3390/nano15020140/s1, Figure S1. (a) fitting of the measured radial diffraction profile of the GaN004 reflection including the individual satellites using the Pseudo-Voigt functions in the software package “Origin Lab”, the dashed lines given as example for SL0 and SL1 correspond to the baseline for the background and therefore the areas of the peaks were determined as the integrated peak area above the baseline (b) simulation of the measured radial diffraction curve by using the Leptos software package “DIFFRACplus LEPTOS 7” from the company Brucker, Karlsruhe, Germany [23] with the assumption of an indium distribution into the individual quantum wells. Table. S1. Qz-positions obtained from fitting of GaN (004) reflections, calculated lattice constant c parameter, and calculated thermal expansion coefficients *α* by using: $c^{'}=c(1+\alpha T)$ for all temperature range.
