# Supplementary material for: In Situ X-Ray Study During Thermal Cycle Treatment Combined with Complementary Ex Situ Investigation of InGaN Quantum Wells
Source: Nanomaterials (Basel). 2025 Jan 17;15(2):140. doi: 10.3390/nano15020140 (PMC11767672; doi:10.3390/nano15020140)
Supplement: Supplementary file 1 [file nanomaterials-15-00140-s001.zip › nanomaterials-3354958-supplementary.pdf]

In Situ X-Ray Study During Thermal Cycle Treatment Combined with Complementary Ex Situ Investigation of InGaN Quantum Wells

Ewa Grzanka*[1,2], Sondes Bauer*[3], Artur Lachowski[1], Szymon Grzanka[1,2], Robert Czernecki[1], Byeongchan So[4], Tilo Baumbach[3,5] and Mike Leszczynski[1,2]

[1] Institute of High-Pressure Physics, Polish Academy of Sciences, Sokolowska 29/37, 01-142 Warsaw, Poland
[2] Top-GaN Ltd, Solec 24/90, 00-403 Warsaw, Poland
[3] Institute for Photon Science and Synchrotron Radiation, Karlsruhe Institute of Technology, Hermann-von-Helmholtz-Platz 1, D-76344 Eggenstein-Leopoldshafen, Germany
[4] Competence Center for III-Nitride Technology, C3NiT-Janzén, Solid State Physics and NanoLund, Lund University, Box 118, 22100 Lund, Sweden
[5] Laboratory for Applications of Synchrotron Radiation, Karlsruhe Institute of Technology, Kaiserstr. 12, 76131, Karlsruhe, Germany

* Correspondence: Sondes.Bauer@kit.edu, elesk@unipress.waw.pl,

Fig. S1 (a) fitting of the measured radial diffraction profile of the GaN004 reflection including the individual satellites using the Pseudo-Voigt functions in the software package "Origin Lab", the dashed lines given as example for SL0 and SL1 correspond to the baseline for the background and therefore the areas of the peaks were determined as the integrated peak area above the baseline (b) simulation of the measured radial diffraction curve by using the Leptos software package "DIFFRACplus LEPTOS 7.9" from the company Brucker, Karlsruhe, Germany [23] with the assumption of an indium distribution into the individual quantum wells

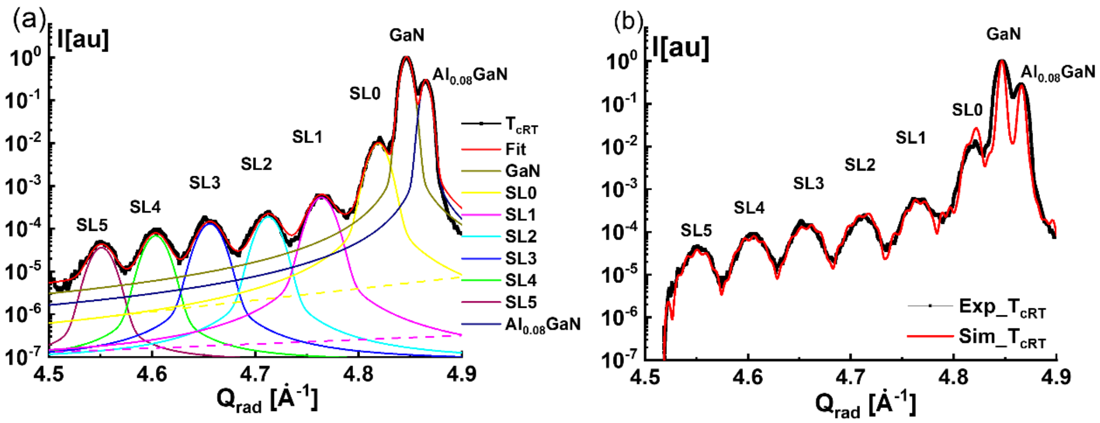

Table. S1 Q_z-positions obtained from fitting of GaN (004) reflections, calculated lattice constant c parameter, and calculated thermal expansion coefficients α by using: $c' = c(1 + \alpha T)$ for all temperature range.

Temperature [°C]	Temperature [K]	Q_z GaN (0004) [Å^{-1}]	c [Å] (4*(2π/Q_z))	α [/K]
RT	298	4.84884	5.1832	3.55E-07
700	973	4.83624	5.1968	2.79E-06
800	1073	4.83424	5.1989	2.91E-06
900	1173	4.83195	5.2014	3.07E-06
920	1193	4.83228	5.2010	2.96E-06
940	1213	4.83201	5.2013	2.96E-06
960	1233	4.83121	5.2022	3.05E-06
980	1253	4.82921	5.2043	3.33E-06
1000	1273	4.82969	5.2038	3.20E-06
